# A geospatial analysis of the impacts of maternity care fee payment policies on the uptake of skilled birth care in Ghana

**DOI:** 10.1186/s12884-016-0833-z

**Published:** 2016-02-29

**Authors:** Fiifi Amoako Johnson

**Affiliations:** Department of Social Statistics and Demography & Centre for Global Health, Population, Poverty and Policy (GHP3), Faculty of Social, Human and Mathematical Sciences, University of Southampton, Southampton, UK

**Keywords:** Maternal health, Geospatial dependence in skilled delivery care, Bayesian geoadditive models, Demographic and Health Survey, Ghana

## Abstract

**Background:**

Many low and middle income countries have initiated maternity fee exemption and removal policies to promote use of skilled maternity care. After two and a half decades of these policies, uptake of skilled birth care remains low and inequalities continue to exist in many low and middle income countries. This study uses 2 decades of birth histories data to examine four maternity fee paying policies enacted in Ghana over the past 3 decades and their geospatial impacts on uptake of skilled delivery care.

**Methods:**

Bayesian Geoadditive Semiparametric regression techniques were applied on four conservative rounds of Demographic and Health Surveys in Ghana to examine the extent of geospatial dependence in skilled birth care use at the district level and their associative relationships with maternity fee paying policies focusing on the temporal trends when the policies were functional.

**Results:**

The results show that at the country-level, the policies had a positive influence on use of skilled delivery care; however their impacts on reducing between-district inequalities were trivial.

**Conclusions:**

The findings suggest that targeted interventions at the district level are essential to strengthen maternal health programmes in Ghana.

## Introduction

Policy actions to reduce the high maternal and neonatal mortality rates in many low and middle income countries have focused on removing financial barriers that limit particularly the poor and marginalised women from seeking skilled maternity care [1–4]. Over the last 3 decades, maternity fee paying policies have ranged from full-fee payment, partial-fee removal, full-fee removal to health insurance schemes [5]. Although governments have invested substantially in fee exemption and removal policies for maternity care, uptake of skilled birth care remains low, inequalities continue to exist and maternal and neonatal mortality continue to be unacceptably high in many low and middle income countries [6]. Despite the enormous financial burden healthcare user-fee exemptions and removal places on developing country governments, proposals for the Sustainable Development Goals (SDGs) for ensuring universal access to health care emphasises removal of user-fees to ensure equity in accessing healthcare.

Till date, there has not been any systematic analysis of the impact of maternity fee paying policies and exemptions on bridging the geographical inequalities in skilled maternity care use. An analysis of this nature is imperative and timely in the context of the post-MDG and SDG agenda aimed at reducing health inequalities through universal access to skilled care. Facility based studies which have often been used to examine the impact of these policies are limited in coverage, rending them ineffective for assessing national-level impact. In addition, many low and middle income countries lack reliable panel data for assessing the temporal effects of such policies. Randomised control trials although are appropriate for establishing causality, scaling-up such efforts at the national level is not feasible. In this study, data from four consecutive (1993, 1998, 2003 and 2008) rounds of the Ghana Demographic and Health Survey (GDHS) are used to examine the extent of geospatial variations in skilled birth care use amongst districts in Ghana. The study further unravels the impact of fee payment policies on the geospatial variations in skilled birth care use focusing on the temporal trends when the policies were functional.

## Background

Over the last 3 decades, Ghana has instituted four major maternity fee payment related policies—the full-cost recovery policy often referred to as “cash and carry” scheme (July 1985–May 1998), free antenatal care policy (June 1998–August 2003), free delivery care policy (initially September 2003–March 2005 for four most deprived regions and April 2005–June 2007 nationally) and the integration of maternity fee payments into the National Health Insurance Scheme (NHIS) (post-June 2007). Under the cash and carry scheme, all health facility users including pregnant women had to pay for full cost of services, even in emergencies before they receive care. This according to research evidence led to increased inequalities in health service use and self-medication [7–11]. To reduce the financial burden of the full out-of-pocket payment for maternity services, the free antenatal care policy was introduced in 1998 under the safe motherhood initiative launched by the Ghana Health Service (GHS) and the Ghana Ministry of Health (MoH) [12]. Under the policy, all pregnant women were exempted from paying fees for antenatal care services in public health facilities. In September 2003, the Government of Ghana secured funding from the Highly Indebted Poor Countries (HIPC) initiative to introduce the free delivery care policy in the four most deprived (Northern, Upper East, Upper West and Central) regions of the country [13]. This policy was rolled out to all regions in April 2005 [14–16]. The policy covered antenatal care, normal deliveries, management of assisted and surgical deliveries.

In 2005 the Government of Ghana introduced the National Health Insurance Scheme (NHIS), which is financed through a 2.5 % levy on value added tax, 2.5 % monthly salary deductions from formal sector workers who are members by default [17, 18] and premium payments ranging from 7.20 to 48.00 Ghana Cedis for informal sector workers [17, 19]. Prior to November 2012, exemptions were provided for children under 18 years with both parents enrolled, the elderly (70 years and older) and the poor classified as the unemployed with no source of income, no fixed residence and not living with someone employed, whose premiums are financed through government budget and donor payments [17–19]. Under the National Health Insurance Act 852, assented by the President of Ghana on 31st October 2012, exemptions were extended to all children irrespective of the registration status of parents, persons with mental disorders and those classified by the Minister for Social Welfare as indigent [20]. NHIS premium holders are eligible to seek medical care from both public and accredited private health facilities [19]. In 2007, the free delivery care policy was ended and maternity care payments were integrated into the NHIS [14, 16]. Pregnant women not registered on the scheme had to pay for maternity services [21]. This led to substantial decline in skilled maternity care use, prompting the government to exempt pregnant women from paying NHIS premiums from July 2008 onwards [16, 21]. Under the exemption, all pregnant women who attended antenatal care at accredited health facilities were registered with the Scheme for a period ending 3 months after delivery [21]. The NHIS does not cover all health service cost. It excludes expensive surgical procedures, cancer treatments, organ transplants and dialysis amongst others [17].

Despite the maternity fee payment policies enacted over the past 3 decades in Ghana, wide geographical inequalities in uptake of skilled birth continue to exists. Over a period of 2 decades (1998 to 2008), uptake of skilled birth care increased from 72 to 84 % in the Greater Accra Region, 51 to 73 % in the Ashanti region but only 2.5 % to only 27.3 % in the Northern region [22, 23]. At the district level, it has been estimated that institutional births range from 7 % in the Yendi district of the Northern region to 85 % in the Ga district of the Greater Accra region [24]. The estimates further show strong geospatial dependence in institutional births [24]. For example, the East Mamprusi district had the highest institutional births of 27 % in the Northern region [24]. Despite the observed wide geospatial variations in use of skilled birth care in Ghana, there are no systematic studies that examine the factors associated with these variations and impact of fee payment policies on bridging these inequalities.

**Fig. 1:**
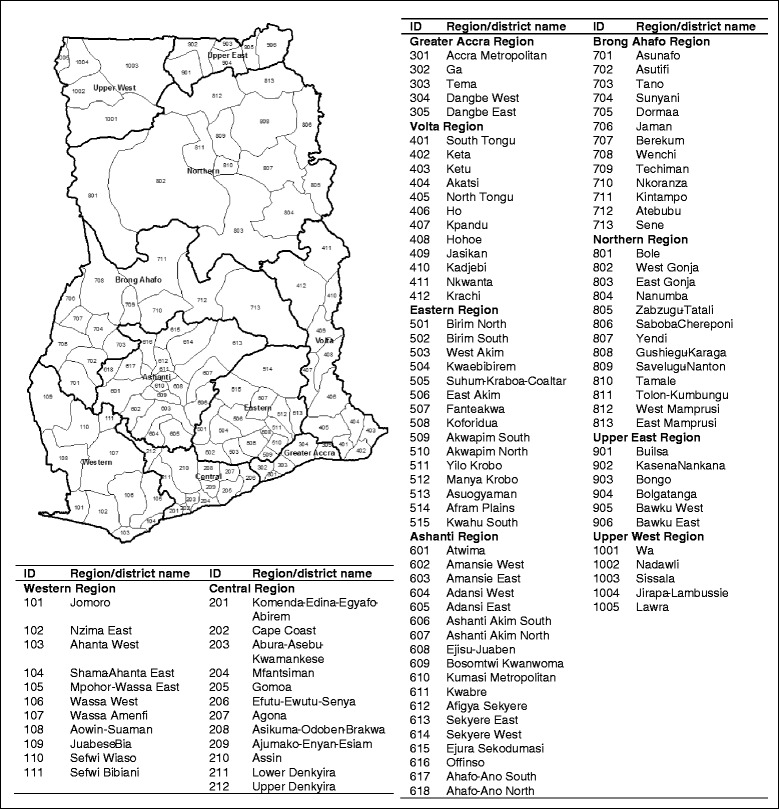
Indexed map of the districts of Ghana

The analysis is conducted at the district level (Fig. 1) because Ghana operates a decentralised system of governance where operation planning, resource allocation and implementation of health services are delivered at the district level [25]. The districts in this study refer to the 110 districts created as part of the political decentralisation of Ghana in 1988 and adopted as the sample frame for the four surveys considered in the analysis.

## Methods

### Data

The data for the analysis comes from four consecutive rounds of the Ghana Demographic and Health Survey (GDHS) conducted in 1993, 1998, 2003 and 2008. The GDHS adopts a two-stage sampling procedure where a systematic sampling procedure is used to select census enumeration areas, also referred to as Primary Sampling Units (PSUs) at the first stage, and households sampled from the selected PSUs at the second stage [22, 23]. GDHS are nationally representative cross-sectional surveys which collect demographic and health information on women, men, children and other members of their household. The 1998, 2003 and 2008 surveys collected information on the place and type of birth care received for all births 5 years preceding each survey, whilst the 1993 survey covered births 3 years preceding the survey. A total of 12,177 births which occurred between November 1990 and October 2008 were covered in the four surveys.

Skilled birth care refers to birth attendants with competency to manage normal deliveries, diagnosis, management of birth complications and referrals [26]. The response variable was binary coded 1 if a birth was attended by skilled health personnel (doctor, nurse or midwife) and 0 otherwise. Since mothers are unlikely to misreport their birth experiences, it is assumed that data on type of birth care are fairly accurate. To minimise recall bias, consistency between reported place of birth and type of birth attendant were examined. Those reported as non-facility births but attended by skilled professionals constitute less than 0.05 % and were excluded from the analysis.

The primary predictor was the timing of births in relation to the periods in which the policies were functional. Given that the data refer to births in the periods during which the policies were operational, if the policies had any measurable impact should reflect in the level of skilled birth care use. The four main maternity fee payment policies enacted between July 1985 and July 2007 were analysed, operationalised as births that occurred during the: (i) “cash and carry” policy (births prior to June 1998); (ii) free antenatal care policy (June 1998 to August 2003); (iii) free delivery care policy (September 2003 to March 2005 for the four most deprived regions and April 2005 to June 2007 nationally) and (iv) the NHIS (births post June 2007). Because of small sample size, the 4 months (July 2008 and October 2008) where pregnant women were exempted from paying NHIS premiums was not analysed separately.

A sensitivity analysis was conducted to examine the extent of dependency (serial correlation) in uptake of skilled birth care across time to ascertain the effectiveness of the temporal structure adopted for the analysis. Autocorrelation (correlogram) techniques were used analyse the dependency in the monthly proportion of births attended by skilled personnel [27]. Since the cash and carry scheme had been in place for 64 months (from July 1985) prior to the start of the window of analysis (November 1990), it is anticipated that its impact on uptake of skilled birth care would have already taken place. To examine how uptake of skilled birth care prior to the free antenatal care, free delivery and NHIS policies were correlated with uptake when the policies came into effect, the births eight months prior to when each policies were implemented were compared to the periods when the policies were in effect.

With regards to the transition from cash and carry to free antenatal care, the analysis revealed positive, highly correlated and statistically significantly (*p* < 0.05) lags before the implementation of the free antenatal care policy when compared to the subsequent lags. Considering the transition from free antenatal care to free delivery care in the four (Central, Northern, Upper East and Upper West) regions, the first seven lags were not statistically significant; however the eight lag was statistically significant. The eighth lag represents the point of transition from free antenatal to free delivery; therefore the significant negative correlation suggest a change in uptake of skilled birth care when the free delivery care was introduced in those regions. This may suggest that structuring the temporal frame from when the policies came into effect were appropriate.

With regards to transition from free antenatal care to free delivery care nationally and from free delivery care to NHIS there were no statistically significant lags. However, the results show stronger negative correlations immediately after the introduction of the policies when compared to the periods before. In this regard, there was no need extending the temporal calibrations to capture specific periods when the policies had an impact. Therefore defining the temporal structure by the period the policies came into effect were appropriate.

The choice of control variables was based on literature and data availability. The selected control variables include maternal age and education, ethnicity, religion, parity, number of antenatal visits, partner’s education, rural-urban place of residence and distance to nearest health facility. Women’s educational status, religion, ethnicity, partner’s educational status and rural-urban place of residence were analysed as fixed (categorical) covariates, whilst maternal age, number of antennal visits, parity and distance to nearest facility were analysed as continuous covariates. To compute the distance to the nearest health facility, a georeferenced list of health facilities that offer maternity services (*n* = 1688) and topographic data on national road-networks were used as input data for a network analysis algorithm to calculate the distance from each Primary Sample Units (PSUs) in the surveys to the nearest health facility using the closest facility functionality in ArcGIS 10.1 [28, 29]. The spatial locations of the health facilities and land surveillance was conducted by the Water Research Institute, the Centre for Remote Sensing and Geographic Information Services (CERSGIS) of the University of Ghana, Department of Feeder Roads of Ghana, Ghana Survey Department and the Forestry Commission of Ghana between 1995 and 2005. The longitude and latitudes values of the PSUs for the four GDHS was provided by ICF International.

Autonomy factors such as needing permission to seek healthcare, who has final say on healthcare decisions and who decides how money is spent were not included in the analysis because they were available for all the surveys. Nonetheless, studies have shown that in Ghana the effect of these autonomy factors on skilled birth care use are trivial when socioeconomic factors such as educational status are accounted for [30].

### Statistical analysis

Differentials in skilled care use aggregated by policy at time of birth and the selected fixed effects (categorical covariates) were examined through bivariate analysis. The Chi-squared test was used to examine significant changes in the uptake of skilled birth care across the policy periods. The mean and quartile distributions of the continuous covariates aggregated by women who received skilled birth care and those who received unskilled birth care were also examined. Bayesian Geoadditive Semiparametric (BGS) regression technique [31] was used to examine the extent of geospatial dependence in skilled birth care use and their associative relationships with maternity fee paying policies focusing on the temporal trends when the policies were functional. To identify the independent effect of the policies, important predictors of skilled birth care use were accounted for based on the literature and data availability. A key advantage of the BGS technique is that it allows for the simultaneous estimation of non-linear effects of continuous covariates as well as fixed effects of categorical and continuous covariates in addition to spatial effects. BGS models also produce maps of the posterior spatial effects, which allows for the impact of the covariates in explaining the spatial patterns of the outcome variable to be examined.

The outcome variable of interest *y*_*ij*_ is coded 1 if woman *i* in district *j* had a skilled birth care and 0 otherwise. In this regard, the outcome variable *y*_*ij*_ follows a binomial distribution with expected probability of skilled birth equal to *π*_*ij*_. The model linking the probabilities of a woman *i* in district *j* having a skilled birth care is the logistic model of the form

1 $$ \left.{y}_{ij}\right|{\eta}_{ij}\sim B\left({\pi}_{ij}\right) $$

2 $$ {\pi}_{ij}=P\left({y}_{ij}=\left.1\right|{\eta}_{ij}\right)=\frac{ \exp \left({\eta}_{ij}\right)}{1+ \exp \left({\eta}_{ij}\right)} $$

where *η*_*ij*_ is the predictor of interest. If we have a vector *x*_*ij*_^'^ = (*x*_*ij*1_, …, *x*_*ijk*_)^'^ of *k* continuous covariates and *λ*_*ij*_^'^ = (*λ*_*ij*1_, … *λ*_*ijd*_)^'^ a vector of *d* categorical covariates, then the predictor *η*_*ij*_ can be specified as

3 $$ {\eta}_{ij}=\alpha {\lambda}_{ij}^{\hbox{'}}+\beta {x}_{ij}^{\hbox{'}} $$

Where *α* is a *d*-dimensional vector of unknown regression coefficients for the categorical covariates *λ*_*ij*_^'^, *β* is a *k*-dimensional vector of unknown regression coefficients for the continuous covariates *x*_*ij*_^'^. The sampling design adopted by the GDHS [23] imposes a clustering effect and introduces dependent observations where use of skilled birth care may not only depend on individual attributes but also community characteristics [32]. Ignoring the hierarchical structure of the data risk overlooking the importance of group effects and may also lead to bias estimation of standard errors, leading to erroneous model results [32]. To account for non-linear effects of the continuous covariates and spatial dependence in skilled birth care use, the BGS framework which replaces the strictly linear predictors with flexible semiparametric predictors was adopted. The BGS model is then specified as shown in Eq. 4 below

4 $$ {\eta}_{ij}=\alpha {\lambda}_{ij}^{\hbox{'}}+{f}_k{x}_{ijk}^{\hbox{'}}+{f}^{spat\left({S}_i\right)} $$

where *f*_*k*_(*x*) are non-linear smoothing function of the continuous variables *x*_*ijk*_ and ${f}^{spat\left({S}_i\right)}$ accounts for unobserved spatial heterogeneity at district *j* (*j* = 1,…,S), some of which may be spatially structured (correlated) and others unstructured (uncorrelated). The spatially structured effects show the effect of location by assuming that areas which are geographically close are more similar than distant areas, whist the unstructured spatial effect accounts for spatial randomness in the model. In this regard, Equation 4 can be specified as

5 $$ {\eta}_{ij}=\alpha {\lambda}_{ij}^{\hbox{'}}+{f}_k{x}_{ijk}^{\hbox{'}}+{f}^{str\left({S}_i\right)}+{f}^{unstr\left({S}_i\right)} $$

where *f*^*str*^ is the structured spatial effects and *f*^*unstr*^ is the unstructured spatial effects and ${f}^{spat\left({S}_i\right)}={f}^{str}+{f}^{unstr}$. In the case of this study, the spatially structured effects depicts the extent of clustering of skilled birth care use and the influence of unaccounted predictor variables that themselves may be spatially clustered or random. The full Bayesian approach, using the Markov Chain Monte Carlo (MCMC) simulation techniques was adopted [33].

Model suitability was assessed using the Deviance Information Criterion (DIC) [34]. The DIC combines a Bayesian measure of model fit with a measure of model complexity to examine model fit. The DIC is based on the posterior distribution of the deviance given by *D* = − 2*logp*(*y*|***θ***), where (*y*|***θ***) is the likelihood of the observed data given the set of parameters ***θ***. If $\overline{D}\left(\boldsymbol{\theta} \right)$ is the posterior mean deviance and $D\left(\overline{\boldsymbol{\theta}}\right)$ is the deviance of the posterior mean, then the effective number of parameters in the model ${P}_D=\overline{D}\left(\boldsymbol{\theta} \right)-D\left(\overline{\boldsymbol{\theta}}\right)$ and the $\mathrm{D}\mathrm{I}\mathrm{C}=\overline{D}\left(\boldsymbol{\theta} \right)+{P}_D$, where $\overline{D}\left(\boldsymbol{\theta} \right)$ accounts for the fit of the model and *P*_*D*_ accounts for the model complexity. Small values of DIC are associated with better models.

A sequential model building approach was adapted to examine how the policies and control factors explain the spatial variations in skilled birth care use across districts. To examine if there exists significant geospatial variation in skilled birth care use, a null model (Model 1) was first fitted then compared to Model 2 which included the spatial effects. Model 3 included the policy periods to examine their impacts on skilled birth care use. The controls were then included in Model 4 to ascertain their effect and also to examine the independent effect of the policies. In Model 4, all continuous variables were fitted as non-linear effects. Only covariates significant at *p* < 0.05 were retained in the model, except for the primary factor upon which the principal research questions of interest was based [32]. The statistical software BayesX was used for the analysis [33].

### Ethical considerations

The GDHS data is available in anonymous format upon request for which no formal ethical approval is required. Ethical approval to conduct the GDHS was obtained from the ICF Macro Institutional Review Board (IRB), Calverton, USA and the Ghana Health Service Ethical Review Committee, Accra, Ghana. Approval was sort from the ICF Macro International to analyse the data.

## Results

### Bivariate analysis

Table 1 shows the percentage of births attended by skilled health personnel aggregated by policy at the time of birth and the selected fixed covariates. Chi-squared test was used to test for significant changes across the policy periods. The results show that, overall the percentage of births attended by skilled personnel increased significantly (*p* < 0.01) over the policy periods. During the cash and carry policy, 44.3 % of births were attended by skilled health personnel, which increased to 49.5 % during the free antenatal care policy and to 54.4 % during the free delivery care policy and to 58.6 % when maternity care was integrated into the NHIS. The results further show that although there was statistically significant increase in uptake of skilled birth care over the policy periods amongst women with no formal education, those with partners with no formal education, women from the poorest households and rural areas, their uptake remains substantially lower when compared to other women (Table 1).

**Table 1:** Percentage distribution of births attended by skilled health personnel aggregated by policy at time of birth and the fixed covariates

Background characteristics	Cash and carry	Free antenatal	Free delivery	NHIS	All
	% (*n*)	% (*n*)	% (*n*)	% (*n*)	% (*n*)
Overall*	44.3 (5056)	49.5 (4671)	54.4 (1612)	58.6 (887)	48.6 (12,226)
Educational background					
No formal education*	24.5 (2220)	31.0 (2084)	30.7 (695)	38.1 (326)	28.6 (5325)
Primary	49.6 (1712)	46.2 (1020)	52.4 (385)	52.7 (208)	49.0 (3325)
Secondary or higher*	67.6 (1124)	69.5 (1567)	78.8 (532)	76.8 (353)	71.0 (3576)
Religious affiliation					
Christian*	52.8 (3290)	55.7 (3144)	62.4 (988)	65.4 (590)	56.0 (8012)
Muslim*	33.9 (681)	41.0 (887)	46.5 (365)	51.5 (189)	40.7 (2122)
Other	20.6 (1085)	20.9 (640)	26.7 (259)	26.2 (108)	21.7 (2092)
Ethnicity					
Akan*	53.9 (2319)	57.8 (1883)	66.4 (560)	67.8 (318)	57.8 (5080)
Ga-Dangbe	59.5 (319)	56.8 (314)	58.3 (60)	58.3 (40)	58.2 (733)
Ewe-Guan*	41.2 (709)	49.9 (670)	60.2 (208)	60.3 (141)	48.2 (1728)
Mole-Dagbane*	21.6 (733)	31.2 (1000)	36.7 (485)	46.9 (234)	30.6 (2452)
Grussi-Gruma-Hausa*	22.4 (623)	28.7 (449)	25.5 (219)	43.0 (123)	26.6 (1414)
Other*	32.9 (353)	45.5 (355)	69.2 (80)	54.5 (31)	44.1 (819)
Partner's educational status					
No formal education*	27.4 (1972)	32.0 (1993)	33.9 (695)	40.5 (351)	31.0 (5011)
Primary*	44.4 (1237)	34.3 (395)	38.7 (135)	47.4 (84)	42.1 (1851)
Secondary or higher*	58.4 (1847)	63.2 (2283)	70.9 (782)	72.7 (452)	63.4 (5364)
Household wealth status					
Poorest*	16.8 (1033)	22.2 (1435)	18.3 (591)	24.3 (292)	20.0 (3351)
Poor*	28.8 (1073)	33.5 (1042)	49.0 (351)	44.8 (185)	34.5 (2651)
Middle*	32.6 (896)	44.8 (859)	63.4 (259)	66.7 (151)	43.6 (2165)
Rich*	52.7 (1016)	71.8 (710)	81.5 (242)	84.2 (156)	64.9 (2124)
Richest*	80.3 (1038)	91.9 (625)	96.3 (169)	97.4 (103)	86.6 (1935)
Place of residence					
Urban*	78.7 (1231)	81.2 (1303)	84.3 (504)	86.6 (297)	81.3 (3335)
Rural*	32.3 (3825)	33.6 (3368)	37.8 (1108)	41.5 (590)	34.0 (8891)

*P < 0.01; n – sample size

With regards to the continuous covariates, the results show that the mean age of women who had skilled birth care is significantly lower than those who had unskilled birth care (Table 2). Also, women who had skilled birth care are significantly likely to be of lower parity, had a higher number of antenatal visits and are in closer proximity to health facilities when compared to those who had unskilled birth care.

**Table 2:** Mean and quartile distributions of the continuous covariates aggregated by women who had skilled and unskilled birth care

Type of birth care received and background characteristics	Mean [95 % CI]	Quartiles		
		1st quartile	2nd quartile	3rd quartile
Skilled birth care				
Maternal age	27.84 [27.6, 28.0]	22.67	27.17	32.50
Parity	3.11 [3.06, 3.17]	1.00	3.00	4.00
Number of antenatal visits	5.48 [5.38, 5.58]	3.00	6.00	8.00
Distance to nearest health facility (km)	3.90 [3.76, 4.05]	0.72	1.97	4.77
Unskilled birth care				
Maternal age	28.43 [28.3, 28.6]	22.83	27.50	33.67
Parity	3.82 [3.88, 3.93]	2.00	3.00	5.00
Number of antenatal visits	3.10 [3.03, 3.17]	0.00	3.00	5.00
Distance to nearest health facility (km)	7.23 [7.07, 7.40]	2.57	4.98	9.50

*CI* confidence intervals

### Multivariate analysis

The estimated posterior odds ratios of skilled birth care use and their 95 % credible intervals for the fixed covariates are displayed in Table 3, along with their model summary statistics. A sequential model building technique was used to analyse the impact of the policies and control variables on use of skilled birth care. Interpretation of the model coefficients is based on the final model (Model 4), since it accounts for the policy periods, controls and the spatial effects.

**Table 3:** Estimated posterior odds ratios of the fixed effects and their corresponding 95 % credible intervals

Variables	Model 1 OR [95 % CI]	Model 2 OR [95 % CI]	Model 3 OR [95 % CI]	Model 4 OR [95 % CI]
Primary variable				
Policy at time of birth				
Cash and Carry			1.00	1.00
Free antenatal care			1.24 [1.13, 1.35]**	1.17 [1.04, 1.31]**
Free delivery			1.92 [1.68, 2.19]**	1.67 [1.42, 1.96]**
NHIS			2.10 [1.79, 2.47]**	1.65 [1.37, 1.99]**
Control variables				
Educational status				
No formal education				1.00
Primary				1.15 [1.01, 1.30]*
Secondary or higher				1.66 [1.43, 1.92]**
Religious affiliation				
Christian				1.00
Muslim				1.00 [0.85, 1.17]
Other				0.56 [0.48, 0.65]**
Partner's educational status				
Don't know/No formal education				1.00
Primary				1.31 [1.13, 1.50]**
Secondary or higher				1.53 [1.36, 1.72]**
Household wealth status				
Poorest				1.00
poor				1.19 [1.03, 1.37]*
middle				1.24 [1.07, 1.44]**
rich				1.42 [1.23, 1.66]**
richest				2.15 [1.84, 2.53]**
Place of residence				
Urban				1.00
Rural				0.33 [0.28, 0.38]**
Model summary statistics				
$\overline{D}\left(\theta \right)$	16,836.2	14,106.4	13,963.4	11,423.5
$D\left(\overline{\theta}\right)$	16,835.2	14,011.9	13,865.2	11,305.1
*P* ^*D*^	1.0	94.5	98.2	118.4
DIC	16,837.2	14,201.0	14,061.7	11,541.8
Posterior mean spatial effects [SD]				
Structured	NA	1.53 [0.26]	1.52 [0.26]	0.66 [0.15]
Unstructured	NA	0.02 [0.02]	0.02 [0.03]	0.02 [0.02]

Model 0: null model—without covariates and spatial effects; Model 1: spatial effects only (no covariates); Model 2: policy at time of birth + spatial effects; Model 3: policy at time of birth + controls + spatial effects

**P < 0.01; *p < 0.05, *CI* credible intervals

#### Geospatial dependence in skilled birth care use

**Fig. 2:**
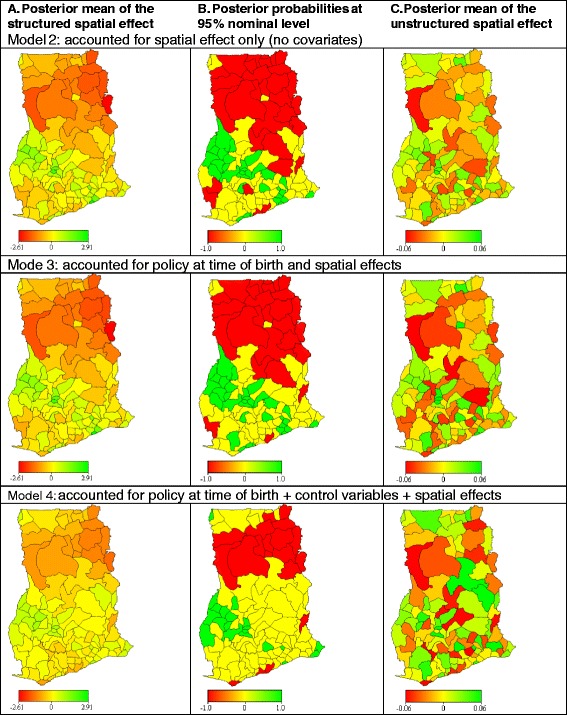
District level **a** structured spatial effects of the posterior mean **b** corresponding posterior probabilities at 95 % nominal level and **c** unstructured spatial effects of the posterior mean. Note: The posterior mean of the structured spatial effects show districts where uptake of skilled birth care are high (*green*), low (*red*) and where uptake an non-uptake are not substantially ifferent (yellow), adjusting for the variables in the model. The posterior probabilities at 95 % nominal level show districts with *statistically significatly* high (*green*) uptake (95 % credible intervals lie in the positive), low (*red*) (95 % credible intervals lie in the negative) and (*yellow*) where they are not significantly different (95 % credible intervals include 0). The posterior probabilities are used to identify the spatial correlations of the covariates with use of skilled birth care by comparing colour changes (*red* to *yellow* or *green* to *yellow*) between models. When a variable(s) is introduced into the model and the posterior probabilities changes from red to yellow or green to yellow, then it implies that the included variable(s) is significantly associated with skilled birth care use in those districts where the colour changes occurred. Also, a cluster of similar colours indicate statistical dependence of the skilled birth care use, as is evident in the north

The estimated DIC for Model 1 (null model) was 16,837.2 (Table 3). When the spatial effects were included in the model (Model 2) the DIC reduced by 2636.2. The high reduction in the DIC when the spatial effects were included in the model shows that births attended by skilled personnel are not spatially randomly distributed but clustered. The estimated posterior mean of the structured spatial effects from the null model (structured spatial effect = 1.53, standard deviatio*n* = 0.26) is substantially higher than the posterior mean of the unstructured spatial effects (unstructured spatial effect = 0.02, standard deviatio*n* = 0.02), further confirming that there is strong spatial dependency in the use of skilled birth care in Ghana. The posterior mean of the structured spatial effect from Model 2 (Fig. 2a) shows districts where without adjusting for any predictors, uptake of skill birth care are low (red) and where they are high (green). The corresponding posterior probabilities at the 95 % nominal level (Fig. 2b) shows districts where skilled birth care use are significantly low (red), significantly high (green) and where they are not significant (yellow). Where the posterior probabilities are not statistically significant, the probability of having a skilled birth care is similar to the probability of not having a skilled birth care. The estimated posterior mean of the structured spatial effects and their corresponding posterior probabilities at the 95 % nominal level (Model 2, Fig. 2a and b) shows a clear north-south divide in use of skilled birth care, with uptake being significantly lower in districts in the northern part (Northern, Upper East and Upper West regions) of the country when compared to those in the south.

#### Factors associated with the geospatial dependence in skilled birth care use

When the policy periods were added to the model (Model 3), the DIC reduced by 139.3, however the estimated posterior mean of the structured spatial effects declined by only 0.01 % (Table 3). This suggests that, although at the national-level the policies had an influence on skilled birth care use, their impact on bridging the between-district inequalities were trivial. At the national-level, the estimated posterior odds ratios from the final model (Model 4) shows that the odds of skilled birth care use increased when the exemption and removal policies became functional (Table 3). The odds of skilled birth care use were 17 % higher during the free antenatal care period, 67 % higher during the free delivery care period and 65 % higher when payment for maternity care services was integrated into the NHIS, when compared to the cash and carry period.

The posterior probabilities at the 95 % nominal level were used to identify the spatial correlations of the covariates with skilled birth care use by comparing colour changes (red to yellow or green to yellow) between models, i.e. examining where the estimated posterior mean of the structured spatial effects (Fig. 2a) becomes statistically non-significant (Fig. 2b) after covariates are added to the model. A comparison of the posterior mean of the structured spatial effects and their corresponding probabilities at 95 % nominal level for Modes 2 and 3, show that the policies were spatially correlated with skilled birth care use only in the Sefwi Wiaso district in the Western region, Mfantsiman district in the Central region, Afram Plains district in the Eastern region and Adansi East district in the Ashanti region. This clearly suggests that the geographical impact of the policies was minimal. It is worthwhile noting that the north-south divide in uptake of skilled birth care remained even after controlling for the policy periods.

**Fig. 3:**
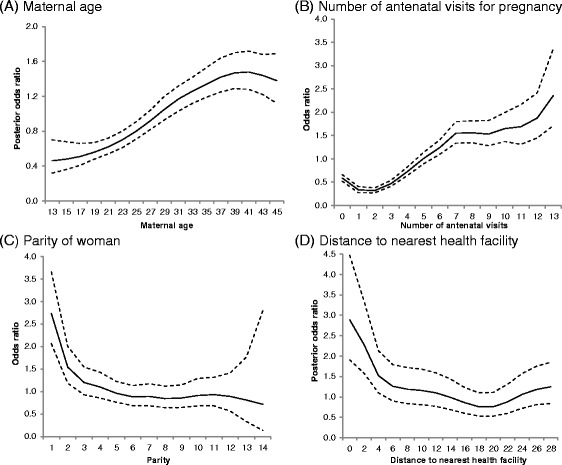
Posterior odds of uptake of skilled birth care and their 95 % credible intervals

When the control variables were included in the model (Model 4) the DIC reduced by a further 2519.9. The estimated posterior odds ratios shows that the policies remained significant even after adjusting for other predictors (Table 3), indicating the independent effect of the policies on skilled birth care use at the national level. The fixed effects (controls) significantly associated with skilled care use at birth were educational status, religious affiliation, partner’s educational status, household wealth status and place of residence (Table 3). The posterior odds ratios presented in Table 3 shows that educated women and those with educated partners are significantly more likely to use skilled birth care. In addition, women from richer households and those in urban areas are significantly more likely to use skilled birth care. Using a flexibly non-parametric modelling approach, non-linear effects of the continuous variables were also examined. The continuous covariates maternal age, parity, number of antenatal visits and distance to the nearest health facility exhibited non-linear association with use of skilled birth care (Fig. 3). The estimated posterior odds ratios show that after adjusting for the variables in the model uptake of skilled birth care increases with increasing maternal age and number of antenatal care visits (Fig. 3a and b), and decreases with increasing parity and distance to nearest health facility (Fig. 3c and d).

After accounting for the controls, the posterior mean of the structured spatial effects decreased by 56.6 %, indicating that the controls are important in explaining some the geospatial variations in uptake of skilled birth care. Although the structured spatial effects reduced substantially, it remained statistically significant (*p* < 0.05), suggesting that the policies and control factors do not explain all the geospatial variations in skilled birth care use amongst districts in Ghana. A comparison of the posterior mean of the structured spatial effects and their corresponding 95 % nominal level probabilities (Fig. 2) for Modes 3 and 4 show that the controls were spatially correlated with low skilled birth care use in the Bawku West, Bongo, Builsa and Kasena-Nankana districts in the Upper East region and the Nadawli and Sissala districts in the Upper West region, where educational levels, antenatal care use and access to health facilities are generally low and poverty and fertility levels are high. These findings were also observed for the East Gonja district in the Northern region, the Atebubu and Sene districts in the Brong Ahafo region, the Nkwanta district in the Volta region and Aowin-Suaman district in the Western region. The controls are positively correlated with use of skilled birth care mostly with districts in the Southern part of Ghana where educational levels, antenatal care use and access to health facilities are generally high and poverty and fertility levels are low. The effects are important in the Adansi West, Ashanti Akim North, Ejisu-Juaben and Sekyere West districts in the Ashanti region, Wenchi and Asunafo districts in the Brong Ahafo region, Cape Coast and Komenda-Edina-Egyafo-Abirem districts in the Central region, Akwapim North, Akwapim South, Kwahu South and Manya Krobo districts in the Eastern region, the Ga and Tema districts in the Greater Accra region and the Shama-Ahanta East and Wassa West districts in the Western region.

The posterior mean of the structured spatial effects and their corresponding probabilities at 95 % nominal level (Model 4, Fig. 2a and b) shows that the north-south divide in skilled birth care use still remained even after adjusting for both the policy periods and controls factors. Uptake of skilled birth care remains significantly low in districts in the Northern region of the country, even after adjusting for the policy periods and the control variables. The districts with significantly low skilled birth care use and not correlated with the selected covariates were the Bole, West Gonja, Nanumba, Zabzugu-Tatali, Saboba-Chereponi, Yendi, Gushiegu-Karaga, Savelugu-Nanton, Tamale, Tolon-Kumbungu, West Mamprusi and East Mamprusi districts in the Northern region. The Ahanta West district in the Western region, the Gomoa district in the Central region, Hohoe district in the Volta region, Wa district in the Upper West region and Bolgatanga and Bawku East districts in the Upper East region also reported low use of skilled birth care which are not explained by selected covariates.

Primarily, in the urban conglomerations, use of skilled birth care was not dependent on women’s demographic and socioeconomic background. For example, districts in the Ashanti (Atwima, Bosomtwi Kwanwoma, Kumasi Metropolitan, Kwabre, Afigya Sekyere and Ahafo-Ano North) and Brong Ahafo (Asutifi, Tano, Sunyani, Dormaa, Jaman, Berekum and Techiman) regions recorded high use of skilled birth care but these are not explicitly explained by the selected covariates. Also, the Juabeso-Bia district in the Western region, Accra Metropolitan in the Greater Accra region, Keta and Ketu districts in the Volta region, Kwaebibirem and Koforidua districts in the Eastern region and Lawra district in the Upper West region also recoded high uptake of skilled birth care but these are not explained by the selected covariates.

## Discussion

Over the last 3 decades, many low and middle income countries have enacted and re-oriented maternity fee exemption policies to bridge the inequality gap in use of skilled maternity care services. This research is the first of its kind at the national level, which uses birth history data to systematically examine the geospatial impact of maternity fee exemption and removal policies on skilled birth care use. The study identified wide geographical differentials in use of skilled delivery care in Ghana. Using repeated cross-sectional population representative survey data covering almost 2 decades of varying maternity care fee policies in Ghana, the study shows that at the national-level the exemption and removal policies had a positive impact on uptake of skilled delivery care when compared to the cash and carry period. This finding concurs with studies that analysed the impact of maternity fee payment policies on use of skilled birth care in Ghana [35]. However, this study has revealed that the impacts of the policies in bridging the between-district inequality gap in skilled birth care use were trivial. At the district level, the results show that considerable geospatial variations continue to exist in the use of skilled birth care with a strong north-south divide, even after adjusting for the policy periods and important demographic and socioeconomic predictors.

The results further show that the between-district posterior structured spatial effects reduced by 56.6 % when the demographic and socioeconomic controls were included in the model. However, the structured spatial effects remained statistically significant, with the posterior probabilities at the 95 % nominal level showing a clustering of districts with significantly low use of skilled birth in the northern part of the country. The findings suggest that the demographic and socioeconomic factors which most national-level analysis have associated with use of skilled birth care, do not explain all the north-south divide amongst districts in Ghana [35–38].

Research evidence shows that user fee payments are not the only financial determinants of seeking skilled maternity care. Out-of-pocket payments for drugs and services not covered under user fee exemptions and removal, transportation cost and unofficial payments are also regressive financial barriers to seeking skilled maternity care [17, 39]. Previous studies has shown that although maternity fee exemption policies has reduce the rich-poor gap in accessing skilled delivery care, the benefits accrued more to the rich when compared to the poor [35]. It has also been reported that the poor often lack information about fee exemptions, and where waivers are available for the poor they have not been effective, because there are often no official records to ascertain eligibility, contributing to low uptake amongst the poor [9, 39–41]. The three northern (Northern, Upper East and Upper West) regions are the most poorly developed regions in the country in terms of poverty incidence and are also characterised by low levels of education, high fertility rates, short birth intervals and high infant and child deaths [22]. Therefore, the observed low uptake of skilled birth care in the northern part of the country could be attributed to other financial barriers not related to official payments for care.

Non-financial barriers have also been identified to hinder women’s decision to seek care, even where services are available. Quality related issues such as long waiting times, provider intolerance, negligence and discrimination as well as impolite attitudes of providers have all been reported to deter women from seeking skilled care [42]. In addition, cultural beliefs, norms and values have also been associated with uptake of skilled maternity care. For example, the belief that obstructed labour is due to infidelity, birth is a test of endurance and care seeking is a sign of weakness has been identified to hinder women’s decision to seek skilled delivery care [43–45]. In some societies, it is culturally unacceptable for a man to be present during delivery, therefore discouraging women from seeking delivery care from male providers, thus avoiding facility births [46, 47]. Other studies have reported that the belief that the placenta should be buried around the house also discourages women from seeking skilled birth care [46].

The northern part of Ghana is particularly culturally oriented and as such culture is strongly associated with behaviour of individuals, including health seeking [48]. Small scale studies in northern Ghana have reported similar quality related issues and cultural constraints to seeking skilled maternity care [48–52]. These factors potentially could explain some of the observed geospatial differentials in skilled birth care use, particularly the low uptake in the northern part of Ghana. However, large scale population level surveys such as the Demographic and Health Survey do not collect such information.

The findings from this study suggests that removal of user fees is essential for improving skilled birth care use, however other barriers need to be addressed to improve uptake for poor and marginalised communities. The evidence from the study shows that there is the need for targeted interventions and strengthening of maternal health programme, particularly amongst districts in the northern part of the Ghana, where it has been shown that the impact of fee exemption and removal policies were trivial.

## Conclusions

Maternity user fee exemption and removal has been one of the key policy strategies to bridge the inequality gap in use of skilled maternity care services in many low and middle income countries. A number of studies have examined the impact of user fees on skilled birth care use at the national level; however, there is no systematic evidence on the impact on bridging the geographical inequalities. This research using repeated cross-sectional population representative survey data spanning 2 decades of four major maternity care fee policies in Ghana has shown that at the national-level fee exemption and removal improved use of skilled birth care. However, the impacts on bridging the between-district inequality gap were trivial. Considerable geospatial variations continue to exist in the use of skilled birth care with a strong north–south divide. Uptake of skilled birth care is particularly low amongst districts in the northern part of the country. The findings from this study suggests that removal of user fees is essential for improving skilled birth care use, however targeted interventions and strengthening of maternal health programmes to address both the financial and non-financial barriers, particularly amongst districts in the northern part of the Ghana are needed to bridge the inequality gap in use of skilled birth care.
